# Gender parity among the Altmetric Top 100 publications on COVID-19

**DOI:** 10.2144/fsoa-2020-0175

**Published:** 2020-11-02

**Authors:** Morteza Mahmoudi

**Affiliations:** 1 Department of Radiology & Precision Health Program, Michigan State University, East Lansing, MI 48824, USA

**Keywords:** Altmetric, coronavirus, gender parity, integrated functioning, research

The current enormous gender disparity in the sciences has drawn considerable attention and sparked widespread debate. Working together, researchers, women scientists who have been discriminated against in scientific recognition and promotions, and supportive journalists and legislators are addressing the question, ‘where are women in the sciences?’ by documenting inequities and exploring root causes. To probe the effectiveness of such efforts in addressing the gender discrepancy, in this paper I analyzed the top 100 Altmetric COVID-19 publications to determine differences between first/last authored male and female papers. The outcomes were striking: the efforts in increasing awareness of the current scientific gender discrepancy among some stakeholders (e.g., researchers, bloggers and journalists) were initially effective, as there were no significant gender discrepancy in Altmetrics scores, news coverage, discussions on blogs and social media coverage.

Unlike conventional scientific metrics (e.g., paper citations), the Altmetrics index is a new way of looking at the impact of scientific papers, providing a wide range of information from various sources, including mainstream media coverage, discussions on blogs and mentions on social media [1]. Analysis using conventional scientific metrics (e.g., number of publications, citations and impact factor) has revealed the effects of gender bias in scientific publications, using the field of chemistry as an example [2]. The current COVID-19 pandemic has the potential to exacerbate this well-documented gender gap in favor of men; for example, a recent survey [3] on the adverse effects of the pandemic revealed that it may unequally affect male and female scientists; more specifically, female scientists with young children had much less time to devote to research compared with others. Taking the publications produced thus far during the COVID-19 pandemic and their associated (social) media coverage as representative of women’s and men’s scientific impacts, the central aim of this Editorial was to investigate whether Altmetrics of COVID-19-related papers also reveal a gender gap. Looking at Altmetric may be useful in defining whether (social) media and blogs cover the news of scientific findings in a gender-specific manner.

I extracted information on the COVID-19 papers with the top 100 Altmetrics scores using Altmetric Explorer (accessed 7 August 2020; see Supplementary Table for details) [4]. It is noteworthy that three manuscripts (two retracted and one editorial with no author information) were excluded from the list; therefore, I extracted the top 103 Altmetric scores. It is also worth mentioning that the online discussions of retractions might contribute to the high Altmetric scores of those retracted papers. The Altmetric Top 100 lists are published annually to provide information on the papers that were discussed most often in mainstream and social media [5]. From the extracted 100 Altmetrics scores of papers on COVID-19, I tallied numbers of men and women among first and last authors. Next, gender-specifics for Altmetrics scores, news coverage, discussions on blogs and mentions on Twitter and Facebook pages were analyzed.

**Figure 1.**
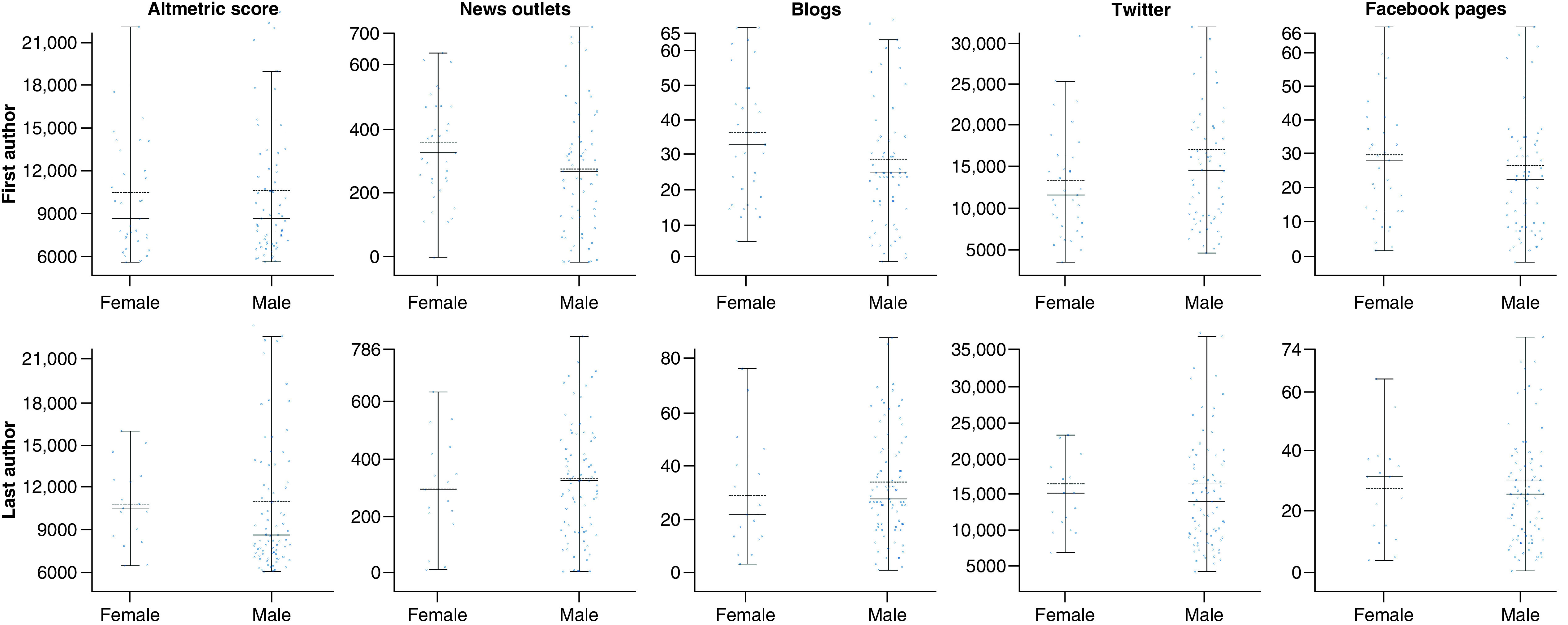
Gender parity among the Altmetric Top 100 publications on COVID-19. Scatter plots showing the Altmetrics scores, news coverage, discussions on blogs and mentions on Twitter and Facebook pages for manuscripts with male and female **(A)** first and **(B)** last authors. Solid and dashed lines show median and mean data (respectively). Statistical analysis (using ANOVA test) of the data revealed that none of the outcomes was significant at p < 0.05. Details of the manuscripts and Altmetric metrics are available in Supplementary Excel file. ANOVA: Analysis of Variance.

The Altmetric Top 100 papers on COVID-19 included: 65 (65%) men and 35 (35%) women as first authors, 83 (83%) men and 17 (17%) women as last authors; and 58 (85%) men and 10 (15%) women as both first and last authors. These findings strongly support the existence of a gender gap in COVID-19 publications, which agrees with other reports [6]. However, for more in-depth analysis of the gender discrepancy beyond the number of papers, I analyzed the Altmetrics scores, news coverage, discussions on blogs and mentions on Twitter and Facebook pages (see Figure 1 for details). Strikingly, those outcomes point to gender parity in the importance of the papers among social media researchers, journalists and the general public.

Working together, researchers and supportive journalists are increasing awareness on the current issues of gender imbalances in science’s backyard [7]. For example, many scientists boycott male-dominated conferences and workshops; and conduct research on gender imbalances and publish their research and experiences in scientific and public forums to raise awareness [7]. These results suggest that the efforts in increasing awareness of the current scientific gender discrepancy among some stakeholders were initially effective, as there were no significant differences between male and female first/last authored papers in terms of Altmetrics scores, news coverage, discussions on blogs and social media coverage in the 100 Altmetric COVID-19 publications. This finding is of great importance as it shows evidence of the significant efficacy of integrated functioning among stakeholders compared with actions by any individual stakeholder. A good example is the recent reports of the National Academies of Sciences, Engineering, and Medicine regarding inefficacy of the existing strong policies and legal recourse in dramatic reduction of the incidence of sexual harassment in academia [8,9]. In other words, in the absence/lack of integrated function among stakeholders, the individual efforts failed to substantially change the culture that fuels sexual harassment.

Following this seemingly successful initial step in improving gender parity in our scientific backyard, one feasible next step would be to address imbalances in the number of publications across male and female scientists, which requires effective and timely action through the involvement of more influential stakeholders (e.g., grant agencies, institutions, editors and decision makers). Gender balance in the number of publications and authorship (specifically first and last authors) is crucial, as women make up a considerable proportion of the scientific, engineering and health workforce [10] (e.g., ∼52% overall and 38% in academics in the USA alone [11]). Ultimately, of course, the only effective way to address the current major ethical issues (e.g., academic incivility, racism and gender imbalances) in our scientific backyard is an integrated functioning between all stakeholders (e.g., researchers, funding agencies and decision makers) in a progressive and efficient manner (more in-depth detail on how such integrated functioning works can be found in our recent view point on academic incivility [12]).
